# Clinical impact of multidrug-resistant bacterial infections in patients with cirrhosis

**DOI:** 10.2144/fsoa-2023-0160

**Published:** 2024-05-14

**Authors:** Nouha Trad, Ghanem Mohamed, Sondes Bizid, Hatem Ben Abdallah, Riadh Bouali, Mohamed Nabil Abdelli

**Affiliations:** 1 Gastroenterology department, Principal Military Hospital of Instruction of Tunis, Faculty of medicine of Tunis, University Tunis El Manar Tunis, Tunisia

**Keywords:** bacterial drug resistance, bacterial infection, liver cirrhosis, mortality, prevalence

## Abstract

**Aim:** Recently, the emergency of multidrug-resistant organisms (MDRO) has complicated the management of bacterial infections (BI) in cirrhosis. We aimed to assess their clinical impact on patients with decompensated cirrhosis. **Methods:** A retrospective study included consecutive cirrhotic patients hospitalized for acute decompensation (AD) between January 2010 and December 2019. **Results:** A total of 518 AD admissions in 219 patients were included, with 260 BI episodes (50.2%). MDRO prevalence was 38.2% of the total isolates. Recent antibiotic use (OR = 4.91), nosocomial infection (OR = 2.95), and healthcare-associated infection (OR = 3.45) were their main risk factors. MDROs were associated with empiric treatment failure (OR = 23.42), a higher prevalence of sepsis (OR = 4.93), ACLF (OR = 3.42) and mortality. **Conclusion:** The clinical impact of MDROs was pejorative, with an increased risk of empiric treatment failure, organ failure and death.

## Plain language summary

In recent years, an increasing spread of multidrug-resistant bacteria had been observed worldwide. The emergence of these strains could represent a major problem in fragile patients such as cirrhotic patients. Over 10 years, our study analyzed the bacteriological profile of cirrhotic patient infections. The multidrug-resistant bacteria prevalence was 38.2%. Exposure to healthcare facilities and recent antibiotic use, were their main risk factors. These strains had a negative impact with an increased risk of treatment failure and death.

## Tweetable abstract

New Study Alert!

Significant prevalence and negative clinical impact of multidrug-resistant organism (MDRO) infections in patients with cirrhosis.

Main risk factors: recent antibiotic use and exposure to healthcare facilities.

#cirrhosis #bacterialinfection #antibiotherapy #resistance.

## Abstract (plain language summary)

Patients with decompensated cirrhosis have a significant rate of multidrug-resistant organism infections.

Recent systemic antibiotic use, nosocomial infection and healthcare-associated infection represent the main risk factors of multidrug-resistant organism infections.

The most isolated multidrug-resistant organisms in cirrhotic patients are mainly Gram-negative bacteria; nevertheless, the infection rate of Gram-positive bacteria is gradually increasing.

Multidrug-resistant organism infections harm cirrhotic patients with increased risk of empiric therapy failure, organ failure and mortality.

Bacterial infection (BI) is a common complication of cirrhosis [1]. Its prevalence can reach 32–40% in patients with decompensated cirrhosis [1]. BI is a serious complication given the increased risk of liver decompensation, organ failure and death [2]. Early diagnosis and rapid prescription of appropriate empiric antibiotic therapy are the basis for the management of this complication [3].

In recent years, an increasing spread of multidrug-resistant organisms (MDRO) had been observed worldwide in healthcare facilities and the community [4]. Thus, the emergency of these strains could represent a major concern in cirrhotic patients [5] who combine several risk factors for colonization by MDROs [6]: recurrent hospitalizations; frequent use of invasive maneuvers; and broad prescription of antibiotic therapy for prophylactic and curative purposes [7,8].

The MDRO resistance profiles differ considerably among geographical areas [7,9]. There is relatively limited data on changing bacterial ecology in developing countries, particularly in cirrhotic patients.

In this study, our objective was to assess the prevalence and risk factors for MDROs in cirrhotic patients hospitalized for acute decompensation (AD) in a North African tertiary center. We also aimed to assess the clinical impact of these strains in decompensated cirrhotic patients.

## Materials & methods

### Study design & participants

This was a retrospective study, including consecutive cirrhotic patients hospitalized in a Tunisian tertiary center for AD between January 2010 and December 2019. The non-inclusion criteria were: under 18 years of age, pregnancy, infection with the human immunodeficiency virus, specific infections (tuberculosis, brucellosis, etc.) and infections requiring urgent surgical treatment (cholecystitis, intra-abdominal abscess, etc.). The setting of care was a regular ward. Patients were transferred to an intensive care unit if necessary.

We collected demographic, clinicals, laboratories and endoscopic data for each admission for AD. Characteristics of the infection, microbiological data, treatment administered (empirical antibiotic therapy and therapeutic escalation) and outcomes were also gathered. Analysis was limited to culture-positive infections. Since we were interested in the short-term prognosis, we studied each infection episode separately regardless of whether it was the first or the n^th^ episode. Two groups have been identified (infection with and without MDROs) to determine MDRO risk factors and to investigate their clinical impact on organ failure and mortality.

### Definitions & outcomes

The diagnosis of cirrhosis was based on histological findings or clinical, biological, radiological and endoscopic features. AD has been diagnosed by the occurrence of one or more major complications of cirrhosis: The development of ascites, hepatic encephalopathy and/or upper gastrointestinal bleeding by rupture of esophageal or/and gastric varices [10].

Infection was considered nosocomial if symptoms of infection appeared 48 h after admission [11]. Infection was classified as healthcare-associated (HCA) if the diagnosis was made on admission or within 48 h of admission in a patient who had previous contact with the healthcare facilities (hospitalization for at least 2 days during 90 days before the infection; residence in a retirement home or long-term care facility; chronic hemodialysis, etc.) [12]. The infection was classified as community-acquired if the symptoms of infection had appeared before admission or within 48 h after hospitalization and if the infection did not have any criteria for HCA [12].

Diagnostic paracentesis was performed in all patients at admission. Spontaneous bacterial peritonitis (SBP) was defined as a neutrophil count in ascitic fluid greater than 250/mm^3^ without any source of intra-abdominal infection treatable surgically [13]. The diagnosis of urinary tract infection (UTI) was based on the combination of a urine leukocyte count >10/mm^3^ with symptoms of urinary irritation, and/or clinical or laboratory signs of infection, with or without a positive culture [14]. Respiratory infection was defined as the combination of respiratory symptoms, typical signs on auscultation, and general signs of infection with alveolar, interstitial or bronchial radiological syndrome [14]. The diagnosis of spontaneous bacteremia was based on positive blood culture without another recognized source of infection [14,15]. Skin and soft tissue infections were defined by clinical signs of infection combined with swelling, warmth, erythema and tenderness of the skin [16]. The other BI were diagnosed according to conventional clinical, laboratory and radiological criteria [17].

Multidrug-resistant (MDR) was defined as acquired non-susceptibility to at least one agent in three or more antimicrobial categories [18]. Extensively drug-resistant (XDR) was defined as non-susceptibility to at least one agent in all but two or fewer antimicrobial categories [18]. Pandrug-resistant (PDR) was defined as non-susceptibility to all agents in all antimicrobial categories [18]. The intrinsic resistance of bacteria was not considered.

In this study, the following bacteria were considered as MDR [7,11]: extended-spectrum beta-lactamase-producing (ESBL) Enterobacteriaceae, methicillin-resistant *Staphylococcus aureus* (MRSA), *Stenotrophomonas maltophilia*, and vancomycin-susceptible enterococci (VSE). The VSE were resistant to ampicillin, penicillin and third-generation cephalosporins. The following bacteria were considered as XDR in the current study [7,11]: vancomycin-resistant enterococci (VRE), carbapenem-resistant Enterobacteriaceae (CRE), carbapenem-resistant *Pseudomonas aeruginosa* and carbapenem-resistant *Acinetobacter baumanii*.

Antibiotic treatment failure was defined as persistent clinical or biological signs, the necessity of therapeutic escalation, or death during treatment.

Acute kidney injury (AKI) was defined as an increase in serum creatinine ≥0.3 mg/dl (≥26.5 umol/l) within 48 h or as a 50% increase from serum creatinine baseline within the last 7 days [13]. The diagnosis of sepsis was based on sepsis-3 criteria [19,20]. The septic shock was defined by sepsis with persisting hypotension requiring vasopressors to maintain mean arterial pressure (MAP) of 65 mmHg or greater and having a serum lactate level >2 mmol/l in the absence of hypovolemia [20]. The diagnosis of acute-on-chronic liver failure (ACLF) was based on the European Association for the Study of the Liver (EASL) consortium for chronic liver failure (CLIF) [21].

### Statistical analysis

In the descriptive analysis, means and standard deviations were calculated for quantitative variables. The categorical variables were expressed as absolute and relative frequencies. In univariate analysis, the student's *t*-test was performed to compare two means over independent series. For categorical variables, Pearson's chi-square test was used to compare percentages on independent series. Univariate analysis followed by multivariate analysis using logistic regression was carried out to identify risk factors of MDROs. The odds ratio (OR) with a 95% CI was used. The significance level for the statistical tests was set at 5%.

### Ethical considerations

Anonymity and confidentiality were respected throughout the study.

## Results

### Study population

**Figure 1.**
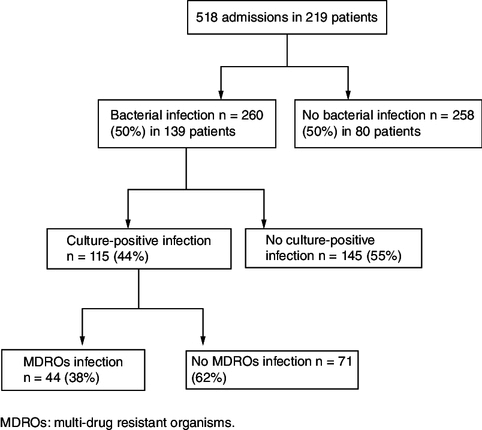
Flow chart of patients included in the study. MDROs: Multidrug-resistant organisms.

A total of 219 cirrhotic patients were included, with a mean age of 61.2 ± 13.06 years and a sex ratio (M/F) of 1.64. Viral cause (57%) was the main etiology of cirrhosis followed by nonalcoholic steatohepatitis (21%). These patients were admitted to our department for AD 518 times, with an average of 2.36 admissions per patient. Figure 1 illustrates the flow chart of included patients.

260 episodes of BI were reported in 139 patients (63.4%) with an average of 1.87 episodes per patient. 66 patients (30.1%) experienced two or more episodes of BI. These episodes were diagnosed in 50.2% of admissions for AD. BI episodes were distributed as follows: 46.87% UTI, 17.57% respiratory infection, 15.9% SBP, 11.7% spontaneous bacteremia, 6.7% skin and soft tissue infections and 1.25% other sites of infection. 39 cases of urinary colonization were ruled out. Microbiological cultures were positive in 115 episodes (44.2%): 74 from urine cultures, 28 from blood cultures, 12 from ascites cultures and one from ear pus culture. Gram-negative bacteria were the most common isolates (74%). Culture-positive BI was classified as community-acquired, HCA and nosocomial in 30.4, 52.2 and 17.4%, respectively. UTI (64.3%) was the most common infection, followed by spontaneous bacteremia (24.3%) and SBP (10.4%). The characteristics of culture-positive BI are summarized in Table 1.

**Table 1.** Comparison of group characteristics according to the presence or absence of multidrug-resistant organisms.

Variable	Total isolates (N = 115)	MDROs (N = 44)	No MDROs (N = 71)	p-value
**Age, years, mean (SD)**	63.9 (12.1)	64.4 (11.6)	63.6 (12.4)	0.724
**Male, N (%)**	69 (60)	27 (61.4)	42 (59.1)	0.486
**Comorbidities**				
Diabetes, N (%)	56 (48.7)	25 (56.8)	31 (43.7)	0.119
arterial hypertension, N (%)	41 (35.6)	17 (38.6)	24 (33.8)	0.371
coronary artery disease, N (%)	11 (9.6)	5 (11.3)	6 (8.4)	0.417
Hypothyroidism, N (%)	9 (7.8)	4 (9)	5 (7)	0.474
**Smoking, N (%)**	45 (39.1)	18 (40.9)	27 (28)	0.454
**Alcohol, N (%)**	6 (5.2)	4 (9)	2 (2.8)	0.150
**BMI, kg/m^2^, mean (SD)**	27.9 (5.4)	27.2 (5.3)	28.2 (5.3)	0.356
**Etiology of cirrhosis:**				
HCV, N (%)	29 (25.2)	9 (20.4)	20 (28.2)	0.755
HBV, N (%)	21 (18.2)	8 (18.1)	13 (18.3)	
NASH, N (%)	24 (20.9)	8 (18.1)	16 (22.5)	
Alcohol, N (%)	6 (5.2)	4 (9)	2 (2.8)	
Others, N (%)				
**HCC, N (%)**	31 (28.7)	13 (29.5)	18 (25.3)	0.389
**CSPH, N (%)**	111 (96.5)	43 (97.7)	68 (95.7)	0.504
**CHILD-Pugh, mean (SD)**	9.5 (2.2)	10.6 (2.2)	8.9 (2)	0.001
**MELD, mean (SD)**	17.9 (5.8)	20.1 (5.6)	16.2 (5.3)	<0.001
**MELD sodium, mean (SD)**	21.7 (6.2)	25.3 (5.3)	19.43 (5.6)	<0.001
**INR, mean (SD)**	1.50 (0.36)	1.45 (0.32)	1.59 (0.41)	0.738
**BILIRUBIN, mean (SD)**	52.4 (72.3)	50.9 (42.9)	70.8 (103.3)	0.022
**Fluoroquinolones prophylaxis, N (%)**	10 (8.7)	5 (11.3)	5 (7)	0.318
**Treatment with Rifaximin, N (%)**	53 (46)	21 (47.7)	32 (45.1)	0.466
**Treatment with PPI, N (%)**	15 (13)	5 (11.3)	10 (14.1)	0.453
**Systemic Antibiotic treatment in previous 3 months, N (%)**	34 (29.6)	22 (50)	12 (16.9)	0.002
**MDROs isolation in previous 6 months, N (%)**	5 (4.3)	2 (4.5)	3 (4.2)	0.637
**Invasive procedures in the previous month, N (%)**	13 (11.3)	5 (11.3)	8 (11.7)	0.606
**Site of infection:**				
UTI, N (%)	74 (64.3)	23 (52.3)	51 (71.8)	0.108
Spontaneous bacteraemia, N (%)	28 (24.3)	13 (9.5)	15 (21.1)	
SBP, N (%)	12 (10.4)	7 (15.9)	5 (7)	
**Types of infection:**				
Community-acquired, N (%)	35 (30.4)	1 (2.3)	34 (47.9)	<0.001
HCA, N (%)	60 (52.2)	31 (70.4)	29 (40.8)	
Nosocomial, N (%)	20 (17.4)	12 (27.7)	8 (11.3)	
**Leukocytes, 10^9^/l, mean (SD)**	7.4 (6.8)	8.1 (6.9)	7.04 (6.8)	0.439
**CRP, mg/l, mean (SD)**	48.9 (48.6)	59.1 (50.5)	42.5 (46.7)	0.085
**Creatinine, μmol/l, mean (SD)**	129.2 (84.7)	167.6 (109.8)	105.4 (52.9)	0.001
**Urea, mmol/l, mean (SD)**	10.3 (7.3)	12.9 (8.6)	8.6 (5.7)	0.006

CRP: C reactive protein; CSPH: Clinically significant portal hypertension; HBV: Hepatitis B virus; HCA: Healthcare-associated; HCC: Hepatocellular carcinoma; HCV: Hepatitis C virus; MDRO: Multidrug-resistant organism; NASH: Nonalcoholic steatohepatitis; PPI: Proton pump inhibitor; SBP: Spontaneous bacterial peritonitis; SD: Standard deviation; UTI: Urinary tract infection.

### Prevalence & risk factors of multidrug-resistant organisms

MDRO prevalence was 38.2% of total isolates (N = 44). The prevalence of MDR and XDR bacteria was 33 and 5%, respectively. PDR Bacteria have not been isolated from microbiological cultures. ESBL *Escherichia coli* was the main isolated MDRO (43%), followed by ESBL *Klebsiella pneumonia* (20%), MRSA (11%), VSE (9%), CRE (7%), *Pseudomonas aeruginosa* (7%) and *Stenotrophomonas maltophilia* (2%).

The comparison between the groups with and without MDROs is shown in Table 1. Comparing the last 5 years (2015–2019) to the first 5 years (2010–2014), the prevalence of MDROs was significantly higher (77.3 vs 22.7%; p = 0.028) (Table 5). MDRO infection was more common when the liver function was more impaired, according to CHILD-Pugh, MELD and MELD-Na scores. Previous systemic antibiotic therapy use for at least 5 days in the past 3 months was more common in the group with MDROs than in the group without. In addition, infection with MDROs was more frequent in nosocomial and HCA infections. In contrast, antibiotic prophylaxis with fluoroquinolones has not been identified as a risk factor for MDROs. On multivariate analysis, recent systemic antibiotic use, nosocomial infection and HCA infection were independent risk factors for MDROs (Table 2).

**Table 2.** Independent risk factors of infection by multidrug-resistant organisms.

Variable	OR	95% CI	p-value
**Types of infection:**			
HCA, N (%)	3.45	1.54–7.70	0.017
Nosocomial, N (%)	2.95	1.07–7.95	0.006
Antibiotic treatment in previous 3 months, N (%)	4.91	2.08–11.58	0.031

HCA: Healthcare-associated; OR: Odds ratio.

### Management, clinical course & outcomes

The empirical antibiotic therapy was started immediately after the bacteriological samples were taken. Third-generation cephalosporins (57.4%) were the most widely used empiric therapy, followed by fluoroquinolones (32.2%), carbapenems (7%), piperacillin-tazobactam (6.1%), amoxicillin and clavulanic acid (4.3%) and metronidazole (4.3%). Cefotaxime (46.1%) and ceftriaxone (11.3%) were the most commonly used third-generation cephalosporins. Empiric antibiotic treatment was prescribed as monotherapy in 88.7% of cases. The mean duration of empiric therapy was 9.60 ± 5.18 days. The empiric treatment failure rate was 37.4% of cases. Antibiotic treatment was escalated in 25.2% of cases. Piperacillin-tazobactam (31%) and carbapenems (44.8%) were the most commonly used second-line antibiotics. The second-line treatment failure rate was 31% of cases. Third-line antibiotic therapy was used in four cases. Carbapenems and vancomycin were the most widely used antibiotic combination as a third-line treatment (three cases). The mean duration of total antibiotic therapy was 13.83 ± 9.98 days. During SBP, albumin infusion was performed in 91.6% of cases. In BI other than SBP, albumin administration was performed in 58.2% of cases.

The prevalence of AKI, sepsis and ACLF was 37.4, 42.6 and 57.4% of cases, respectively. ACLF grade 1 (36.5%) was the most common grade followed by grade 2 (12.2%), and grade 3 (8.7%). Septic shock was diagnosed in 11.3% of cases. The in-hospital and 28-day mortality rate was 15.2 and 28.3%, respectively.

### Clinical impact of multidrug-resistant organisms

Table 3 illustrates the clinical impact of MDRO infection in cirrhotic patients. MDROs were significantly associated with an increased risk of failure of empiric therapy and more frequent therapeutic escalation. Antibiotic therapy and hospital stays were longer in case of MDRO infection. The occurrence of sepsis, ACLF and AKI was significantly more common during MDRO infection. The intra-hospital and day-28 mortality rate was also significantly higher during this infection (Table 4).

**Table 3.** Clinical impact of multidrug-resistant organisms in cirrhotic patients with acute decompensation.

Variable	Total isolates (N = 115)	MDROs (N = 44)	No MDROs (N = 71)	OR	95% CI	p-value
Failure of empiric therapy, N (%)	43 (37.4)	34 (77.3)	9 (12.7)	23.42	8.67–63.22	<0.001
Escalation of antibiotic treatment, N (%)	29 (25.2)	25 (56.8)	4 (5.7)	22.03	6.82–71.15	<0.001
Duration of antibiotic therapy, day, mean (SD)	13.8 (9.9)	18.1 (14.3)	11.8 (4.9)	–	–	0.035
Length of hospital stay, day, mean (SD)	18.8 (17.8)	23.1 (21.9)	16.3 (14.5)	–	–	0.049
AKI, N (%)	43 (37.4)	27 (61.3)	16 (22.5)	5.46	2.39–12.44	<0.001
Sepsis, N (%)	49 (42.6)	29 (65.9)	20 (28.1)	4.93	2.19–11.08	<0.001
ACLF, N (%)	66 (57.4)	33 (68.2)	33 (46.5)	3.42	1.41–8.27	0.004
Septic shock, N (%)	13 (11.3)	8 (8.2)	5 (7.1)	2.93	0.89–9.63	0.067

ACLF: Acute-on-chronic liver failure; AKI: Kidney injury; MDRO: Multidrug-resistant organism; OR: Odds ratio; SD: Standard deviation.

**Table 4.** Impact of multidrug-resistant organisms on short-term mortality in cirrhotic patients with acute decompensation.

Variable	Total patients with positive culture (N = 46)	Patients with MDROs (N = 18)	Patients without MDROs (N = 28)	OR	95% CI	p-value
In hospital mortality, N (%)	7 (15.2)	6 (33.3)	1 (3.5)	13.50	1.46–124.73	0.005
28 day mortality, N (%)	13 (28.3)	11 (61.1)	2 (7.1)	20.42	3.64–114.35	<0.001

MDRO: Multidrug-resistant organism; OR: Odds ratio.

**Table 5.** Epidemiological evolution of bacterial infection in cirrhotic patients.

Variable	Total	First 5 years (2010–2014)	Last 5 years (2015–2019)	p-value
Admissions, n (%)	518	214 (41.3)	304 (58.7)	–
Infections, n	260	88 (33.9)	172 (66.1)	<0.001
Positive-culture infections, n	115	31 (27)	84 (73)	0.025
MDROs infections, n	44	34 (77.3)	10 (22.7)	0.028

MDRO: Multidrug-resistant organism.

## Discussion

Our study investigated the epidemiological patterns and clinical impact of MDRO infection in cirrhotic patients hospitalized for AD. A remarkable finding was that the prevalence of MDRO was 38% of the total isolates. This finding gives us an idea of the spread of MDROs in cirrhotic patients in North African countries. This prevalence was relatively close to the global prevalence (34%), as reported by the intercontinental Study by Piano *et al.* [7]. Nevertheless, a manifest geographic disparity was noted in this intercontinental study; the MDRO prevalence was higher in Asia (50%) than in Europe (28%) and North America (27%). Among Asian centers, India had the highest prevalence (73%) [7]. This difference in the MDRO prevalence between European and Asian centers was also confirmed by other studies [11,22,23].

ESBL Enterobacteriaceae, mainly *E. coli* and *K. pneumonia*, were predominant in almost all previous studies [7,11,23,24]. However, the share of gram-positive cocci represented by MSRA and VSE or VRE has increased significantly in recent years worldwide [24], particularly in European and American centers [11,25,26]. This trend can be explained by the increased use of invasive maneuvers in these centers [6]. In the present study, ESBL Enterobacteriaceae were the most isolated MDROs (64%). However, the prevalence of multidrug-resistant Gram-positive cocci was, otherwise, not negligible (20%).

Previous use of systemic antibiotics, recent exposure to healthcare facilities, and HCA or nosocomial infections were the MDRO risk factors most reported. Our results were fully consistent with previous findings in this area [7,8,11]. Indeed, antibiotics exert a pressure-selective effect resulting in the elimination of sensitive strains and therefore the speedy growth of multi-resistant strains. On the other hand, healthcare facilities facilitate the spread of these strains among patients and the transmission of drug-resistance genes between bacteria. The high frequency of HCA infections (52%) and of the recent systemic antibiotics use (30%) may explain the quite high prevalence of MDROs in our study.

Furthermore, some previous studies have suggested that SBP prophylaxis with fluoroquinolones has a major role in MDRO spreading [27,28]. However, SBP prophylaxis with fluoroquinolones was not identified as a risk factor for MDROs in this study. Although the number of patients taking this prophylaxis is too small to draw firm conclusions, our results were consistent with the findings of the intercontinental study and with a recent randomized controlled study that compared SBP prophylaxis by fluoroquinolones with placebo in decompensated cirrhosis [7,29]. Thus, the EASL guidelines still recommend the prescription of fluoroquinolones for primary or secondary SBP prophylaxis [13].

The identification of the MDRO risk factors as well as the particularities of the local bacterial ecology are crucial to optimize empirical antibiotic treatment. Indeed, in line with previous studies [7,22,30], our study confirmed the negative impact of MDRO infections in cirrhotic patients. MDRO infections were strongly associated with an increased risk of empirical treatment failure, more frequent recourse to therapeutic escalation as well as prolongation of the antibiotic therapy and hospitalization duration [7,11,23]. Its prognosis was more compromised than the BI with sensitive strains, with a high risk of sepsis, ACLF [15], AKI, and septic shock [7,30,31]. All these elements had, thus, contributed to a potential increase in mortality [32]. Intra-hospital and 28-day mortality was quite higher in MDRO infections [7,11,30,33].

The EASL guidelines published in 2018 recommended relatively narrow-spectrum empirical antibiotic therapy for the treatment of community-acquired infection in cirrhotic patients [13]. Nevertheless, given the spread of MDROs as well as their prognostic impact, the use of a broader spectrum of antibiotic therapy, such as carbapenems and glycopeptides, should be strongly considered in the case of nosocomial infection, HCA infection, sepsis, or ACLF [13]. In real-life medical practice, according to the intercontinental study, the empirical treatment prescribed in the different centers included, adhered to EASL guidelines in only 61% of cases [7]. The efficacy of antibiotic therapy was significantly higher in patients who received treatment that complied with EASL guidelines than in those who did not [7]. Our work was a critical study of our empirical prescriptions in cirrhotic patients by comparing them with the EASL recommendations. An interesting observation must be mentioned; in community-acquired infections, our prescription adhered to EASL guidelines in the majority of cases, whereas in nosocomial and HCA infections, the spectrum of empirical treatment administered was relatively narrower than the guidelines. However, most of the BI included were managed before the publication of these guidelines since our series retrospectively studied BI between January 2010 and December 2019. Finally, we suggested that our current practice is changing and that our prescribing is becoming more and more adherent to these guidelines.

Our study has certain limitations. This was a retrospective and single-center study. Given the heterogeneous geographical distribution of multidrug-resistant strains among centers, our findings should be generalized with caution.

Finally, we insist on good management of antibiotics and compliance with universal rules of hygiene to limit the spread of these multi-resistant strains. we also propose to carry out active screening on admission, by nasal and rectal swabs, of colonization by MDRs in cirrhotic patients [34], in particular in the presence of risk factors [33,35]. The fight against self-medication and the anarchic use of antibiotics in other areas is also required.

## Conclusion

Our study emphasized the significant prevalence of MDRO infections in cirrhotic patients. Recent antibiotic use and exposure to healthcare facilities were their main risk factors. These infections compromised the prognostic of cirrhotic patients with increased risk of empiric therapy failure, organ failure and mortality. Further efforts should be made to control the spread of these virulent strains both in healthcare facilities and in the community.
